# Hematological and gastroprotective effects of *Madhulai Manappagu* syrup in indomethacin-induced gastric ulcer animal models

**DOI:** 10.6026/973206300214102

**Published:** 2025-11-15

**Authors:** Manavalan T, Priyadharshini S, Kalvimoorthi V, Senthilvel G, Gopi L, Sridevi Sangeeth G.K, Meenakshi Sundaram M

**Affiliations:** 1 Department of Pharmacology, Mayor Radhakrishnan College of Pharmacy, Cuddalore, India; 2 Department of Paediatrics (Kuzhandhai Maruthuvam), National Institute of siddha, Chennai-Affiliated to The Tamil Nadu Dr. M.G.R. Medical University, Chennai, India; 3 Department of Pharmaceutics, Aadhi Bhagwan College of Pharmacy, Randam, Thiruvannamalai, Tamil Nadu, India; 4 Department of Gunapadam, National Institute of siddha-Affiliated to The Tamil Nadu Dr. M.G.R. Medical University, Chennai, India; 5 Department of Pharmacology, Faculty of Allied Health Science, Meenakshi Academy of Higher Education, Chennai, India

**Keywords:** Gastric ulcer, hematological parameters, *Madhulai Manappagu*, ulcer index, siddha medicine

## Abstract

Gastric ulcer is a major gastrointestinal disorder with high global prevalence. *Madhulai Manappagu*, a Siddha herbal syrup preparation, is traditionally prescribed for gastric ailments, is of interest to evaluate the gastroprotective and hematological effects of Madhulai Manappagu in indomethacin-induced gastric ulcer in Wistar albino rats. Hematological parameters including hemoglobin, red blood cells, mean corpuscular volume, mean corpuscular hemoglobin, mean corpuscular hemoglobin concentration, packed cell volume, platelet count and serum iron were determined using standard protocols and for anti-ulcer evaluation, *Madhulai Manappagu* was given orally at 500 and 1000 mg/kg body weight. Treatment with *Madhulai Manappagu* significantly (p < 0.00001) and dose-dependently improved hematological indices and serum iron levels in ulcerated rats compared to positive control. *Madhulai Manappagu* demonstrates significant gastroprotective and hematological benefits in indomethacin-induced ulcerated rats hence show its potential as a safe plant-based therapeutic option for gastric ulcer, particularly in bleeding-associated conditions.

## Background:

Peptic ulcers (PU) are lesions of the gastrointestinal (GI) mucosa that extend through the mucosa and may involve the muscular layer. The global prevalence of PU is estimated at 8.4%, with rates as high as 40% in developed nations and 80% in developing countries [1, 2]. Ulcers most commonly occur in the upper duodenum, lower esophageal sphincter, or fundic region of the stomach [3]. Non-steroidal anti-inflammatory drugs (NSAIDs) such as Indomethacin are known ulcerogenic agents due to their inhibition of prostanoid synthesis, which is essential for mucosal defense and repair [4]. Additional factors including gastric acid, pepsin, *Helicobacter pylori* infection, smoking, alcohol intake and stress contribute to ulcer pathogenesis [5]. *Madhulai Manappagu* is a Siddha herbal syrup formulation traditionally prescribed for ulcer and anemia-related conditions in newborns, pregnant women and menstruating individuals. It has been widely used for more than a decade in the treatment of *Veluppu Noi* (iron deficiency anemia), morning sickness, nausea, vomiting and burning sensations in extremities [6]. The formulation improves hemoglobin levels and supports both maternal and fetal health. GC-MS profiling of *Madhulai Manappagu* revealed the presence of sugars, volatile compounds, vitamins, fatty acids, alkanes, nucleosides and mercaptans [7]. Ascorbic acid, an important constituent, enhances intestinal iron absorption and supports erythropoiesis, explaining its efficacy in anemia management [8]. Furthermore, pomegranate-derived compounds exhibit strong antioxidant and anti-inflammatory properties, providing additional protection against gastric mucosal injury [9]. Therefore, it is of interest to investigate the anti-ulcer potential of *Madhulai Manappagu* using an Indomethacin-induced gastric ulcer model in male Wistar rats. Hematological parameters, ulcer indices and histopathological features were evaluated to establish the gastroprotective efficacy and therapeutic applicability of this Siddha herbal syrup.

## Materials and Methods:

## Ethical approval and authentication:

The study protocol was approved by the Institutional Animal Ethics Committee (IAEC) of Aadhi Bhagawan College of Pharmacy (Approval No: 1696/PO/a/13/CPCSEA) in accordance with CPCSEA guidelines. The raw ingredients of *Madhulai Manappagu* (pomegranate, sugar candy, honey and rose water) were authenticated by the Siddha Central Research Institute (Central Council for Research in Siddha, Ministry of AYUSH, Chennai).

## Preparation of *Madhulai Manappagu* syrup:

The formulation was prepared at the Gunapadam Laboratory, National Institute of Siddha, Tambaram Sanatorium, Chennai. Fresh pomegranate juice (500 ml) was combined with rose water (500 ml) and sugar candy (500 g) and boiled until syrup consistency was obtained. After cooling, honey (500 ml) was added and mixed thoroughly before storing in airtight containers [10].

## Physicochemical evaluation: 

The prepared syrup was evaluated for color, taste, odor, viscosity, pH and weight per ml.

## Glucose diffusion assay:

The assay was performed using dialysis bags containing 1% syrup extract with 25 ml glucose solution (20 mmol/L) dialyzed against 200 ml distilled water at 37°C in a shaker bath. Dialysates were sampled at 30, 60, 120 and 180 min and glucose levels were quantified spectrophotometrically. A blank control was included.

## Stability testing:

Syrup samples were stored in amber vials at 40°C, room temperature and 47°C. At 24, 48 and 72 h, samples were analyzed for turbidity, homogeneity and physicochemical parameters.

## Acute toxicity study:

Acute oral toxicity was performed in male Wistar albino rats following OECD guideline 423. Animals were fasted for 4 h with free access to water. The syrup (5 mg/kg, p.o.) was administered and mortality was monitored for 24 and 72 h.

## Animals and experimental design:

Thirty male Wistar albino rats (8 weeks old, 220-260 g) were randomized into five groups (n=6) for a 28-day study:

[1] Group I (Control): CMC (0.5% w/v, p.o.).

[2] Group II (Ulcer control): Indomethacin (500 mg/kg, p.o.).

[3] Group III (Standard): Indomethacin (500 mg/kg) + Pantoprazole (40 mg/kg, p.o.).

[4] Group IV (Test low dose): Indomethacin (500 mg/kg) + *Madhulai Manappagu* (500 mg/kg, p.o.).

[5] Group V (Test high dose): Indomethacin (500 mg/kg) + *Madhulai Manappagu* (1000 mg/kg, p.o.).

## Statistical analysis:

All values were expressed as mean ± SEM. Data were analyzed by one-way ANOVA followed by Tukey's post hoc test. A p-value <0.05 was considered statistically significant.

## Results:

The prepared *Madhulai Manappagu* syrup was brown in color, aromatic in odor and sweet in taste. The pH of the formulation was measured as 7.11, indicating near neutrality. The viscosity of the syrup was found to be 24.58 centipoise, confirm its stable consistency and results shown in Table 1 (see PDF). The diffusion of glucose across the dialysis membrane was found to decrease progressively in the presence of *Madhulai Manappagu* syrup. At an initial glucose concentration of 5 µg/mL, the diffusion value was 0.172, which gradually reduced with increasing concentrations, reaching 0.121 at 100 µg/mL. This indicates that the syrup effectively inhibited glucose movement in a concentration-dependent manner, suggesting potential in controlling postprandial glucose diffusion (Table 2 - see PDF) [11]. The preliminary phytochemical screening of *Madhulai Manappagu* confirmed the presence of alkaloids, carbohydrates, proteins, phenols, flavonoids and sterols, while terpenoids, tannins, glycosides, saponins and fats/oils were absent (Table 3 - see PDF and Figure 1 - see PDF). The presence of alkaloids is considered beneficial, as they exhibit antimicrobial, analgesic and gastroprotective properties [12]. Carbohydrates and proteins are essential for nutritional support and may contribute to the energy-yielding and restorative roles of the formulation [13]. Phenolic compounds and flavonoids are well-known antioxidants that play a critical role in reducing oxidative stress and enhancing mucosal defense, thus providing anti-ulcer activity [14, 15]. Sterols are associated with anti-inflammatory and membrane-stabilizing effects, which may further contribute to ulcer protection [16]. The absence of tannins may be advantageous, as high tannin content can sometimes impair nutrient absorption and cause gastrointestinal irritation [17]. Similarly, the absence of saponins may reduce the risk of hemolytic toxicity associated with high concentrations [18]. The lack of terpenoids, glycosides and fats/oils suggests that the formulation is less likely to cause unwanted lipid accumulation or toxicity but still retains strong therapeutic constituents. Thus, the phytochemical profile of *Madhulai Manappagu* indicates a favorable balance of bioactive compounds with potential hematinic, antioxidant and gastroprotective roles. The stability of *Madhulai Manappagu* syrup (Table 4 - see PDF) was assessed under different storage conditions (5°C, room temperature and 47°C) for up to two months. At 1 day, 1 month and 2 months, the syrup retained its characteristic brown color, aromatic odor and sweet taste across all tested conditions. The pH remained stable at 7.11 initially and showed only a marginal reduction to 7.10 at the end of two months, while the weight per ml remained consistent (1.1835-1.1931). No turbidity or loss of homogeneity was observed in samples stored at 5°C or room temperature throughout the study. Similarly, samples stored at 47°C maintained homogeneity without evidence of precipitation or degradation. These results confirm that *Madhulai Manappagu* exhibits good physicochemical stability under accelerated storage conditions, consistent with reports that herbal formulations containing natural sugars and phenolic compounds demonstrate stable profiles when stored at controlled temperatures [19, 20]. Indomethacin-induced gastric ulceration (Negative control, Group II) produced a marked reduction in hematological indices compared to the normal control group (Group I) (Table 5 - see PDF, Figure 2 - see PDF and 3 - see PDF). Significant decreases were observed in RBC count (5.088 ± 0.02 x10^6^/µL), hemoglobin (11.51 ± 0.19 g/dL), MCH (16.30 ± 0.03 pg), MCV (58.71 ± 0.03 pg), MCHC (35.09 ± 0.03 g/dL) and PCV (31.12 ± 0.04%), reflecting anemia and hematological imbalance associated with ulceration. The standard treatment group receiving pantoprazole (Group III) exhibited partial recovery, with values approaching normal. In comparison, animals treated with *Madhulai Manappagu* showed a dose-dependent improvement. The low dose (500 mg/kg, Group IV) produced moderate increases in RBC, Hb and related indices, whereas the high dose (1000 mg/kg, Group V) significantly restored hematological parameters. Notably, RBC count (7.382 ± 0.05 x10^6^/µL), Hb level (12.82 ± 0.09 g/dL) and MCHC (41.22 ± 0.07 g/dL) in the high-dose group were significantly higher (p < 0.0001) compared to the negative control and were comparable to the standard treatment group. These findings suggest that *Madhulai Manappagu* mitigates indomethacin-induced hematological alterations, possibly due to its iron-rich pomegranate base, ascorbic acid-mediated enhancement of iron absorption and flavonoid/phenolic antioxidant activity. Previous studies have shown that pomegranate extracts improve hemoglobin levels and erythrocyte indices in anemia and ulcer models, supporting the present observations [21, 22]. Administration of indomethacin in the negative control group (Group II) resulted in marked ulcerogenic effects, as evidenced by a significant increase in ulcer score (37.83 ± 1.01) and ulcer index (2.817 ± 0.01) compared to the normal control (Group I), which showed no lesions (Table 6 - see PDF, Figure 2 - see PDF and 3 - see PDF). This was associated with reduced platelet count (606,514 ± 45,037 L/µL) and iron value (15.91 ± 0.03 g/dL), indicating compromised hematological balance and mucosal integrity. Treatment with pantoprazole (Group III, standard control) significantly reduced the ulcer score (8.25 ± 0.44) and ulcer index (0.699 ± 0.01), corresponding to a 72.70% inhibition of ulcer formation. Similarly, animals treated with *Madhulai Manappagu* showed dose-dependent gastroprotective activity. The low-dose group (500 mg/kg, Group IV) exhibited an ulcer score of 20.41 ± 0.27, ulcer index of 1.152 ± 0.01 and 45.50% inhibition, alongside improvements in platelet count (825,742 ± 5,092 L/µL) and iron levels (17.38 ± 0.03 g/dL). The high-dose group (1000 mg/kg, Group V) showed greater efficacy, with a reduction in ulcer score (11.67 ± 0.33) and ulcer index (0.827 ± 0.01), corresponding to 67.67% inhibition, which was comparable to the pantoprazole-treated group. Additionally, platelet (845,762 ± 19,326 L/µL) and iron (18.13 ± 0.03 g/dL) levels were significantly improved compared to the indomethacin group. These findings indicate that *Madhulai Manappagu* confers protective effects against indomethacin-induced gastric mucosal damage, likely through its bioactive constituents including polyphenols, flavonoids and tannins in pomegranate and honey, which are known to exert antioxidant, cytoprotective and mucosal defense-enhancing effects. Earlier studies have documented the antiulcerogenic potential of pomegranate extracts, attributing their protective action to modulation of oxidative stress and enhancement of gastric mucosal resistance [23, 24].

Figure 4 (see PDF) illustrates the antiulcer efficacy of *Madhulai Manappagu* (MM) against indomethacin-induced gastric lesions in rats. The ulcer score (I) and ulcer index (J) were significantly elevated in the negative control group, indicating severe mucosal damage. Treatment with pantoprazole (standard) markedly reduced both parameters, demonstrating its potent gastroprotective effect. Notably, MM supplementation showed a dose-dependent reduction in ulcer severity. The low dose (500 mg/kg) decreased the ulcer score and index moderately, whereas the high dose (1000 mg/kg) exhibited a more pronounced effect, nearly comparable to pantoprazole. The percentage inhibition of ulcer formation (K) further confirmed these findings, where MM at 1000 mg/kg achieved a 67.67% inhibition compared to 72.70% by pantoprazole, while the low dose achieved 45.50% inhibition. This highlights the potential of MM as a natural antiulcer agent with significant mucosal protective activity. The protective effect is likely attributable to the synergistic presence of bioactive compounds in pomegranate and honey, which possess antioxidant, anti-inflammatory and cytoprotective properties that strengthen gastric mucosal defense mechanisms. These results are consistent with earlier reports where pomegranate extracts demonstrated significant gastroprotective activity by reducing ulcer index and enhancing antioxidant defense in NSAID-induced ulcer models [24, 25]. Histopathological analysis (Figure 5 - see PDF) revealed normal gastric mucosal architecture in the control group (Group I), with intact epithelial lining and absence of inflammatory changes. In contrast, the indomethacin-treated group (Group II) displayed severe mucosal disruption, extensive necrosis of the gastric epithelium, glandular destruction and marked infiltration of inflammatory cells, confirming ulcer induction. The pantoprazole-treated group (Group III) showed considerable restoration of gastric tissue integrity, with reduced necrosis and preservation of mucosal barrier function. Rats administered MM at low dose (500 mg/kg; Group IV) exhibited partial protection, characterized by decreased ulceration, reduced epithelial loss and moderate inflammatory infiltration compared to the indomethacin group. Importantly, the high-dose MM group (1000 mg/kg; Group V) demonstrated substantial gastroprotection, with nearly normal mucosal appearance, minimal necrosis, diminished inflammatory infiltration and clear evidence of mucosal barrier restoration. These findings support the histological evidence of MM's protective role against NSAID-induced gastric injury. Macroscopic evaluation of rat gastric tissues (Figure 6 - see PDF) further corroborated the histological findings. Control animals (Group I) had normal stomach morphology, whereas indomethacin-treated rats (Group II) exhibited extensive hemorrhagic lesions and ulcerative spots on the gastric surface. Pantoprazole treatment (Group III) reduced the number and severity of visible lesions, while MM-treated groups showed a dose-dependent protection. At high dose (1000 mg/kg), MM-treated rats (Group V) presented minimal mucosal injury, comparable to the pantoprazole group, suggesting potent ulcer-healing activity of the syrup. These results align with previous studies reporting that pomegranate-based formulations exert gastroprotective effects by enhancing mucosal defense, reducing oxidative stress and suppressing inflammatory responses in gastric ulcer models [25, 26].

## Discussion:

The present study demonstrates the beneficial effects of *Madhulai Manappagu* (MM) on hematological parameters, iron metabolism and gastric ulcer indices in indomethacin-induced ulcerated rats. One of the key findings was the significant restoration of red blood cell (RBC) levels following MM treatment. As expected, indomethacin administration (Group II) led to a marked reduction in RBC count compared to the normal control, consistent with the hematotoxic and ulcerogenic effects of NSAIDs [27]. However, MM-treated groups, particularly the high-dose group (1000 mg/kg, Group V), exhibited a significant increase in RBC count, suggesting that MM may counteract NSAID-induced suppression of hematopoiesis and maintain optimal oxygen-carrying capacity. In parallel, hemoglobin (Hb) levels were considerably reduced in the indomethacin group, reflecting impaired oxygen transport due to gastric mucosal damage and anemia secondary to ulceration [28]. Treatment with MM significantly improved Hb concentrations, with the higher dose producing effects comparable to pantoprazole. This hematinic effect may be attributed to the iron- and vitamin-rich profile of MM ingredients such as pomegranate and honey, which are traditionally used to manage anemia and restore vitality [29]. Mean corpuscular hemoglobin (MCH) and mean corpuscular volume (MCV) values were also decreased in the indomethacin group, indicating the development of microcytic, hypochromic anemia. MM supplementation significantly improved both parameters, restoring RBC morphology and hemoglobin content. These findings suggest that MM ameliorates NSAID-induced alterations in erythrocyte indices, thereby preserving erythrocyte functionality [30]. Importantly, serum iron levels, which are crucial for hemoglobin synthesis and erythropoiesis, were significantly reduced in ulcerated rats. MM treatment, particularly at 1000 mg/kg, markedly elevated serum iron concentrations, approaching values observed in the control group. This suggests that MM may enhance iron absorption and utilization, consistent with the known bioavailability-enhancing effects of ascorbic acid and polyphenols present in pomegranate-based formulations [29]. In addition to its hematinic activity, MM exhibited potent gastroprotective effects. Indomethacin administration significantly increased ulcer score and ulcer index, confirming severe gastric mucosal injury. MM treatment produced a dose-dependent reduction in both ulcer parameters, with the higher dose demonstrating protection comparable to pantoprazole. The gastroprotective activity of MM may be attributed to the antioxidant, anti-inflammatory and cytoprotective actions of its bioactive components, which collectively restore the mucosal barrier and accelerate ulcer healing [30]. Overall, these findings highlight MM as promising Siddha herbal syrup with dual hematinic and gastroprotective potential, making it particularly valuable in conditions where gastric ulceration is accompanied by blood loss and anemia.

## Conclusion:

*Madhulai Manappagu* (MM) exerts significant gastroprotective activity against indomethacin-induced gastric ulcers in rats is shown. Its beneficial effects on hematological parameters-including restoration of RBC count, hemoglobin concentration, mean corpuscular hemoglobin (MCH), mean corpuscular volume (MCV) and serum iron levels-highlight its dual role in both ulcer healing and hematinic support. The combined improvement in ulcer indices and hematological markers suggests that MM not only protects the gastric mucosa but also counteracts the anemia commonly associated with NSAID-induced gastric injury. Further studies are warranted to isolate and characterize the active phytoconstituents of *Madhulai Manappagu* and to validate its efficacy through molecular investigations and clinical trials.

## Abbreviations:

HB: Hemoglobin; RBC:Red Blood cells; MCV:Mean corpuscular volume; MCH:Mean corpuscular Hemoglobin; PCV:Packed cell volume; MCHC: Mean corpuscular Hemoglobin concentration; MM: Maadhulai Manapaggu.
